# How we approach the use of sirolimus and new agents: Medical therapy to treat vascular anomalies

**DOI:** 10.1002/pbc.29603

**Published:** 2022-03-06

**Authors:** Kristin A. Shimano, Whitney Eng, Denise M. Adams

**Affiliations:** 1 Division of Allergy, Immunology, and Bone Marrow Transplant, UCSF Benioff Children’s Hospital, University of California, San Francisco, California, USA; 2 Division of Hematology/Oncology, Dana-Farber/Boston Children’s Cancer and Blood Disorders Center, Harvard Medical School, Boston, Massachusetts, USA; 3 Division of Oncology, Comprehensive Vascular Anomalies Program/Frontier Program, Children’s Hospital of Philadelphia, Philadelphia, Pennsylvania, USA; 4 Division of Pediatrics, University of Pennsylvania School of Medicine, Philadelphia, Pennsylvania, USA

**Keywords:** AKT inhibitor, MEK inhibitors, PIK3CA inhibitor, sirolimus, thalidomide, vascular anomalies

## Abstract

Vascular anomalies (VAs) are a heterogeneous group of primarily congenital tumors and malformations. The International Society for the Study of Vascular Anomalies (ISSVA) has developed a standard classification of these disorders, creating a uniform approach to their diagnosis. Recent discoveries evaluating the genetic causes of VAs have revealed that they are due to mutations in cancer pathways, including the PI3K/AKT/mTOR and RAS/MAPK/MEK pathways. These discoveries have led to improved phenotype–genotype correlation and have expanded medical therapy for this group of unique disorders.

## 1 | INTRODUCTION

Vascular anomalies (VAs) comprise a spectrum of diseases that are broadly classified as tumors and malformations. Diagnosis can be challenging due to overlapping clinical features. The International Society for the Study of Vascular Anomalies (ISSVA) first developed a classification system in 1996 to provide a standard approach to identification and naming of these heterogeneous disorders, and since that time has provided updates to the classification scheme to reflect advances in our clinical and genetic understanding of these disorders. Use of standard terminology has improved communication among patients and providers and provides a framework for accurate diagnosis and treatment. The care of patients with VAs necessitates an interdisciplinary approach to diagnosis, treatment, and follow-up. Improved diagnostics have been a focus of VA centers worldwide, and a collaborative approach is critical to optimize patient outcomes.

Historically, treatment of VAs was primarily surgical and interventional, with limited medical therapies. The medical therapies that did exist were used for a wide array of VAs without systematic trials, making evaluation of efficacy difficult. Recent discoveries highlighting causative genomic mutations have broadened our medical therapeutic options and significantly changed the quality of life of patients. Trials are in development to further investigate mechanism of action, safety, efficacy, and response. In this article, we present three illustrative clinical cases and provide an overview of medical therapy options and the rationale for use in specific diseases.

## 2 | MANAGEMENT APPROACH

Medical therapy is usually considered for complicated VAs that are life threatening, obstruct vital structures, cause significant cosmetic problems, or interfere significantly with a patient’s quality of life. Patients with complex VAs are ideally evaluated by an interdisciplinary VA clinic for accurate diagnosis and creation of a treatment plan by a group that includes expertise of hematologists/oncologists, dermatologists, surgical specialists, radiologists, pathologists, geneticists, and genetic counselors, in collaboration with basic, translational, and clinical researchers.

**FIGURE 1:**
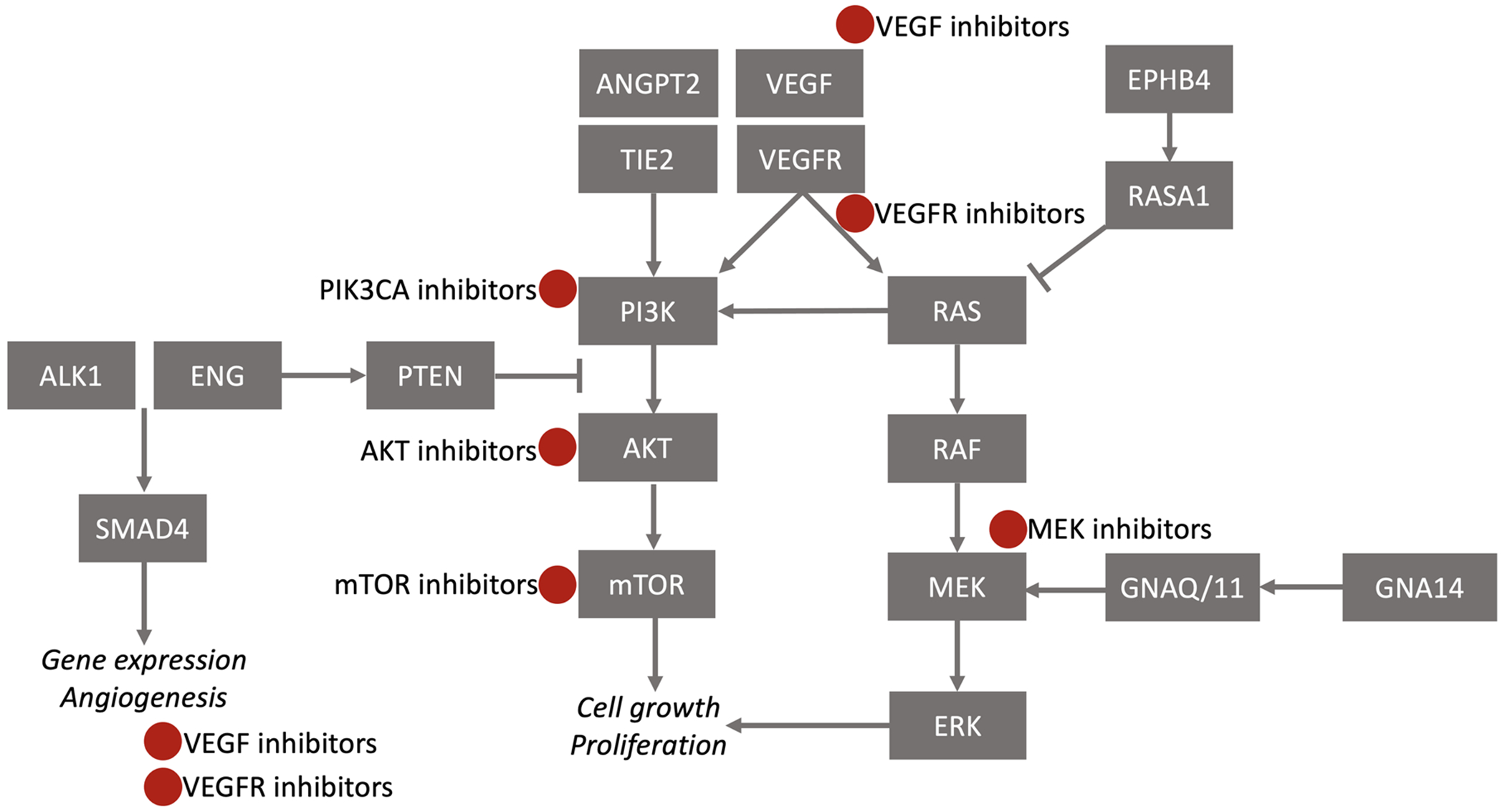
Signaling pathways and potential for targeted therapies in vascular anomalies

Due to recent advances in molecular genetics and decreased cost of sequencing, genetic testing is now becoming part of the diagnostic approach. Many VAs are due to somatic mutations in targetable pathways (Figure 1), and improved phenotype–genotype correlation has expanded options for medical therapy for these patients (Table 1). Prior to the initiation of therapy, however, the team needs to consider many aspects of care, including assessment of phenotype and genotype, eligibility for clinical trials versus off-label treatment, determination of response, and safety monitoring. If a patient’s genotype is unknown, considerations include whether a mutation would provide options for targeted therapeutics, whether germline or somatic testing is appropriate, and, if biopsy of affected tissues is required, how this might be optimally and safely obtained. Treatment decisions should consider available therapies, prior data, reported experience of the agent’s use in this population, and whether a particular medication may be obtained via clinical trial, commercially, or through a compassionate use program. Because there are limited therapies available and few open clinical trials, many medications are being used off-label. As a result, it is important for treating physicians to understand how to deliver these medications safely. The use of off-label and investigational therapies should be directed by a VA specialist.

## 3 | CONSULTATION WITH A VASCULAR ANOMALIES CLINIC

The cases below highlight the complexity of patients with VAs and illustrate the need for interdisciplinary management, standards of practice, and outcomes of studies and clinical trials. Informed consent for the inclusion of representative photographs was obtained from patients or their guardians.

### 3.1 | Case 1

**FIGURE 2:**
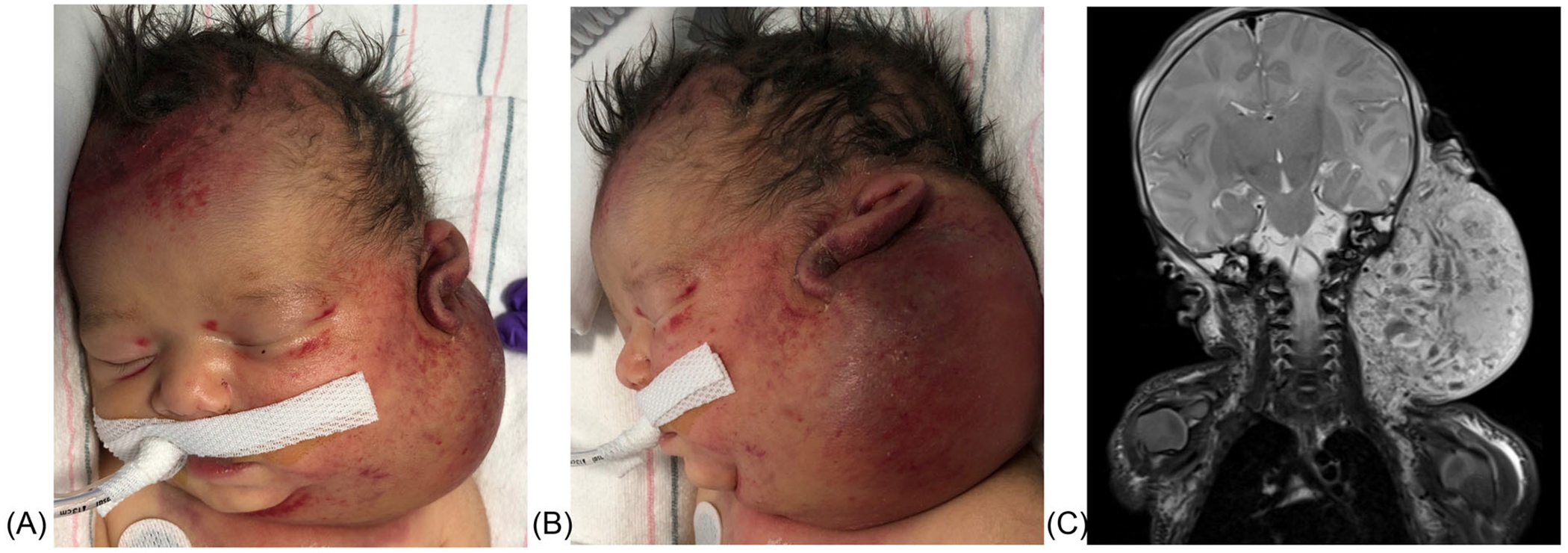
Kaposiform hemangioendothelioma (KHE). (A) There is a large, firm, and indurated mass extending over this infant’s left ear, neck, and cheek. Note the petechiae and purpuric discoloration overlying the skin in this patient with thrombocytopenia and hypofibrinogenemia. (B) The lesion was present at birth and involves the skin, parotid gland, sternocleidomastoid muscle, and the deep spaces of the neck. This patient was intubated due to concern for mass effect of the lesion compressing the patient’s airway. (C) Coronal T2 weighted magnetic resonance imaging (MRI) with fat saturation demonstrates a highly infiltrative mass extending from the left scalp and periauricular region inferiorly into the upper anterior chest wall. The mass crosses fascial planes and extends along the paravertebral muscles and retropharyngeal space. There is also involvement of the nonarticular portion of the left temporomandibular joint, which appears deformed and possibly subluxed

A newborn male presents at birth with a large, purpuric, firm, indurated mass over his left ear, neck, and cheek (Figure 2). Magnetic resonance imaging (MRI) reveals an infiltrative vascular lesion crossing tissue planes and impinging on the airway. His platelet count is 3 × 10^9^/L and his fibrinogen is undetectable. Due to this classic presentation, you recognize that he has kaposiform hemangioendothelioma (KHE) and Kasabach–Merritt phenomenon (KMP). Although there is no standard of care, you are aware that this patient is high risk secondary to age, location, and KMP, and you consider combination therapy with steroids and sirolimus. There are no active clinical trials.

### 3.2 | Case 2

**FIGURE 3:**
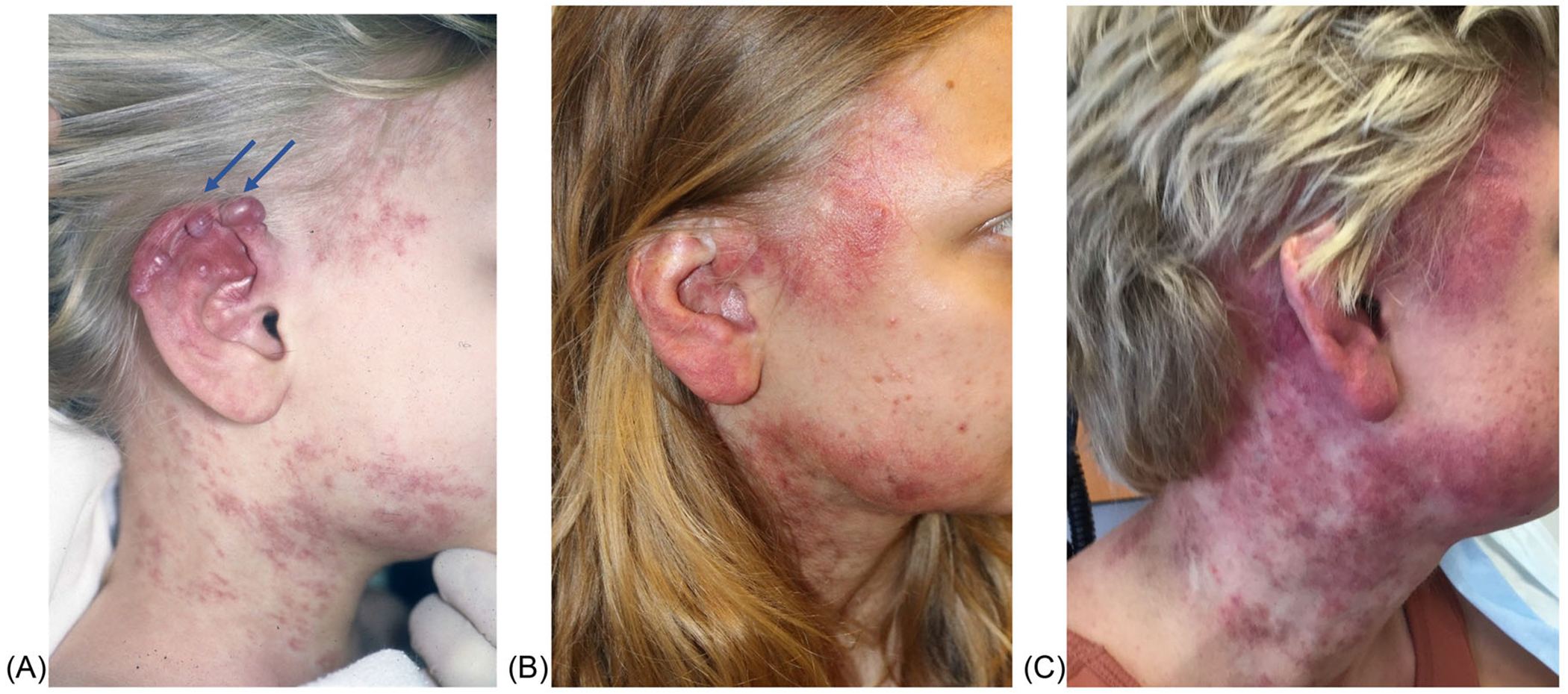
Arteriovenous malformation (AVM) of the scalp, ear, and neck. AVMs are fast-flow malformations that demonstrate significant progression over time. (A) The patient at 4 years of age. The lesions denoted by the blue arrows represent pyogenic granulomas, which often overlie the AVM and have a propensity to bleed. (B) By 21 years of age, the patient’s malformation has extended further, with collateralization of vessels and worsening dilatation of arteriovenous shunting. (C) At 23 years of age, on trametinib, with stable symptoms and requiring fewer procedures overall

A 22-year-old with an extensive arteriovenous malformation (AVM) of the scalp, ear, neck, and face is seen in your interdisciplinary clinic (Figure 3). She has undergone numerous sclerotherapy and embolization procedures, as well as surgical debulking, with significant complications, including a stroke, bleeding, and need for a temporary tracheostomy. Her symptoms are progressive and are severely affecting her quality of life. She is referred to your center after genetic testing elucidated a somatic MAP2K mutation. You consider use of an oral MEK inhibitor, but recognize that there is limited published information, and no open clinical trials available close to the patient. Your options include obtaining this medication commercially or obtaining an IND through the sponsor and FDA.

### 3.3 | Case 3

**FIGURE 4:**
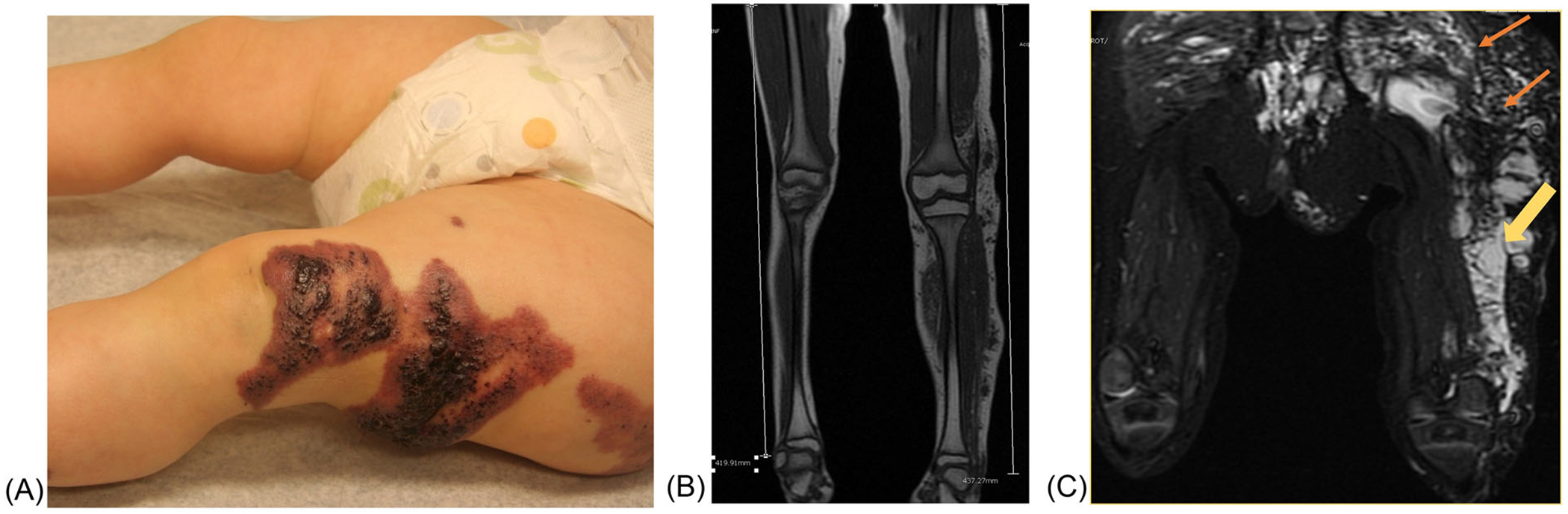
Klippel–Trenaunay syndrome (KTS). (A) KTS or capillary lymphatic venous malformation of the left lower extremity. This disorder is now classified under the genotypic-based term of PIK3CA-related overgrowth syndrome (PROS). Diffuse lymphatic blebs extend over the patient’s leg and perirectal area. These areas are prone to bleeding, leaking, and infection. (B) Limb length discrepancy and subcutaneous changes along the lateral margin of the left lower extremity are noted on T1-weighted magnetic resonance imaging (MRI). (C) Diffusely abnormal soft tissue thickening in the left upper lateral and gluteal compartment demonstrating lymphatic malformation (orange arrows). There are also multiple areas of phlebectasia seen along the lateral compartment of the thigh (yellow arrow)

A child initially presents at birth with a large capillary lymphatic venous malformation (CLVM) of her left lower extremity and bilateral buttocks (Figure 4). At 3 weeks of age, she develops cellulitis and *Escherichia coli* bacteremia. Due to overgrowth, cellulitis, lymphatic blebs, and functional impairment, she is treated with the mammalian target of rapamycin (mTOR) inhibitor sirolimus. She undergoes sclerotherapy procedures to treat large embryonal veins and debulking procedures of overgrown tissue. She does very well until age 4, when she starts having frequent viral illnesses and recurrent episodes of cellulitis of her buttock area. Due to side effects from sirolimus and inadequate therapeutic response, additional treatment options are discussed. After interdisciplinary review, a decision is made to obtain genotyping and consider treatment with a targeted oral PIK3CA inhibitor. You are able to obtain this medication through a managed access protocol, NCT04085653.

## 4 | THERAPEUTIC TREATMENT OPTIONS FOR VASCULAR ANOMALIES

### 4.1 | Sirolimus

Sirolimus, also known as rapamycin, is a specific and potent inhibitor of mTOR, a serine/threonine kinase in the phosphoinositide-3-kinase (PI3K)/AKT pathway. This pathway regulates numerous cellular processes, including cellular catabolism and anabolism, cell motility, angiogenesis, and cell growth.^1^ Sirolimus was first trialed as a treatment for VAs in an infant with KHE and severe KMP, whose disease was refractory to all treatment regimens (steroids, vincristine, cyclophosphamide, bevacizumab, and embolization therapy). The patient exhibited complete resolution of coagulopathy within 2 months of sirolimus initiation and experienced substantial improvements in pain, lesion size, and function.^2^ This initial success prompted the prospective phase II clinical trial assessing the safety and efficacy of sirolimus in the treatment of complicated VAs. The trial resulted in an overall response rate of 83% at 6 months and 85% at 1 year of treatment. Limited toxicity was seen. Significant response was noted in microcystic and complex lymphatic malformations, CLVM, PTEN-hamartoma tumor syndrome, venous lymphatic malformations, and KHE. Responses were noted in clinical symptoms and quality of life regardless of whether improvement or stable disease was noted radiologically.^3^ Since these early successes, the use of sirolimus has rapidly expanded and appears efficacious in a variety of vascular tumors and malformations.^4^ Sirolimus has also been safely used in combination with surgery and interventional procedures, and this combined approach has led to improved overall patient outcomes.^5–8^ Dosing, monitoring, and supportive care practices may vary based on institutional standards and patient specifics, but principles for management include the following guidelines.

Dosing: In children, particularly in neonates and infants, close monitoring is essential given differences in drug elimination, at least in part due to developing organ functions.^9^ In the phase II clinical trial, sirolimus was administered orally as a liquid suspension at an initial dose of 0.8 mg/m^2^ per dose at 12-hour intervals and titrated to maintain a goal serum trough level of 10–15 ng/ml.^3^ Most patients in that study were treated with levels of 10–13 ng/ml. This goal drug level was based on the use of sirolimus in pediatric renal transplant. However, lower sirolimus doses have been efficacious in patients with VAs. As a result, many providers now titrate to lower trough levels, with a goal of minimizing side effects. Dosing and drug levels are based on severity of disease and goals of treatment. Higher trough levels (10–13 ng/ml) are used in the initial treatment of complicated (high-risk) Vas, such as KHE with KMP, Gorham–Stout disease (GSD), generalized lymphatic anomaly (GLA), kaposiform lymphangiomatosis (KLA), and high-risk PIK3CA-related overgrowth syndromes (PROS). Patients with intermediate-risk disease (significant manifestations of disease but no risk for immediate life-threatening complications) can be treated with levels ranging from 6 to 10 ng/ml, while those with low-risk disease (such as single-site lymphatic malformations with minor symptoms) or patients who have already achieved good response at higher doses can be treated with levels of 2–6 ng/ml. Depending on the age of the patient and individual pharmacokinetic differences, some patients may achieve these troughs with daily dosing, while others may require twice daily dosing. A 12-hour trough is followed with BID dosing and a 24-hour level with daily dosing. Adult dosing is 2 mg per day for uncomplicated VAs.^10^ For maintenance therapy, the goal of treatment is to provide the lowest dose with the greatest effect.

Dosing for infants and young children has been studied and recently reported with data from the noted phase II study.^11^ Importantly, neonates (0–1 month of age) require a lower starting dose of 0.4–0.45 mg/m^2^/dose administered twice daily (target trough concentration levels between 10 and 15 ng/ml) due to the decreased CYP3A enzymes that are found in the liver and intestine in this age group, and drug levels should be monitored early (several days after initiation) and frequently to avoid supratherapeutic levels. Most monitor every 3 days until a stable dose is reached.

Because of the heterogeneity of these disorders, a more individualized approach to treatment has been implemented. At the start of therapy, it is important to note the severity of disease, the goal trough level (12 or 24 hour), frequency of labs, and follow-up and timing of response assessment.

Side effects and immunosuppression: Common side effects, which are generally dose dependent, include headaches, gastrointestinal discomfort, mouth sores, elevated triglycerides and cholesterol levels, acne, lymphedema, and bone marrow suppression. Rare but important and/or life-threatening side effects include an increased risk of infections, ovarian cysts, hyperglycemia, problems with wound healing, capillary leak syndrome, pneumonitis, and possible male infertility. Frequent monitoring is necessary while on sirolimus therapy to ensure adequate drug levels and to monitor for toxicities. Suggested monitoring frequency ranges from every 1 to 3 months, depending on the target sirolimus trough levels.

One of the most concerning side effects of sirolimus is immunosuppression. The degree of immunosuppression caused by sirolimus in the treatment of VAs is unclear, and formal testing in one series showed negligible immunosuppression due to sirolimus.^12^ However, many VAs, particularly “leaky” lymphatic anomalies such as KLA and central conducting lymphatic anomalies (CCLA) are associated with protein loss, lymphopenia, and hypogammaglobinemia. Furthermore, infants with VAs on sirolimus who already have immature immune systems are presumed to be at higher risk. Formal testing for immune dysfunction, including lymphocyte subsets, immunoglobulin levels, and lymphocyte stimulation testing, is suggested in these high-risk patients.

The approach to febrile illnesses in patients on sirolimus depends on patient-specific factors and may differ by institutional practice. Patients should be instructed to call their prescribing physician with illnesses. For patients with uncomplicated VAs, no prior history of severe infection and no history of neutropenia on routine monitoring labs, they may be managed expectantly with close follow-up and consider holding sirolimus until the acute illness is resolved. For patients with a history of bacteremia or other severe infections, or those who have had sirolimus-induced neutropenia, immediate evaluation potentially including bloodworks and antibiotics is indicated for fevers. Fevers and illness can slow the metabolism of sirolimus causing higher blood levels.^13^

Patients being treated with sirolimus are at risk for opportunistic infections. This includes the development of *Pneumocystis jirovecii* pneumonia (PJP). PJP has been reported in patients receiving sirolimus after solid organ transplantation and in at least one pediatric patient with a VA.^14^ Presently, it is suggested that those patients at high risk (under 2 years of age, or with evidence of CD4 lymphopenia, trough levels of greater than 10 ng/ml, or receiving concurrent therapy with steroids) should be placed on PJP prophylaxis based on institutional guidelines. It is less clear if older patients or those with lower goal trough levels need PJP prophylaxis.

Vaccination guidelines with sirolimus use are not established, particularly in patients with VAs. It is unclear what effect sirolimus has on the immunity induced by vaccines. Live vaccines should be administered prior to the start of sirolimus, if possible, and avoided while on active treatment with sirolimus. The appropriate timeframe between stopping sirolimus and administration of MMR and varicella vaccines is not established; approaches vary and should be discussed with the local VA team and immunology. Non-live or killed vaccines are safe and recommended in patients on sirolimus therapy. However, the need for titer monitoring and booster immunizations is unclear at this time.^15^

Impaired wound healing is a side effect of sirolimus, but decisions about the perioperative management of the medication should consider the specifics of the surgery; in some cases, continuing sirolimus through the surgical period may decrease swelling and improve the postoperative course. For patients undergoing debulking surgeries, or for those for whom sirolimus is controlling airway lesions, it is often preferable to continue the sirolimus.

Duration of therapy: The optimal duration of sirolimus for treatment of VAs is undefined, differs from patient to patient, and in many cases may be indefinite. Additional longitudinal data are needed to better counsel patients and families on effects of long-term treatment with sirolimus.

### 4.2 | AKT inhibitors

Targeting proteins upstream of mTOR is another attractive strategy for the treatment of patients with VAs that are driven by mutations in genes at many points in the PI3K/AKT pathway. AKT inhibition has been shown to have efficacy in vitro on patient-derived cells from patients with hemihyperplasia multiple lipomatosis (HHML), congenital lipomatosis, overgrowth, vascular malformations, epidermal nevi, spinal/skeletal anomalies, scoliosis (CLOVES), and megalencephaly capillary malformation syndrome (MCAP).^16^ Additionally, endothelial cells from patients with CLVM demonstrated constitutive phosphorylation of AKT, and the proliferation of these cells was inhibited by both AKT and PIK3CA inhibitors.^17^ A patient with Proteus syndrome and a causative somatic AKT mutation was successfully treated with the AKT inhibitor miransertib, with stable disease on imaging and clinical improvement.^18^ A phase I/II clinical trial is ongoing for miransertib in patients with PROS and Proteus syndrome (NCT03094832). This study is active but not recruiting new patients.

### 4.3 | PIK3CA inhibitors

BYL719, an oral PIK3CA inhibitor, is currently FDA-approved for use in patients with PIK3CA-mutated breast cancer. Given that CLOVES is due to somatic mutations in PIK3CA and belongs to the spectrum of PROS, BYL719, was first used in a patient with a severe form of CLOVES in 2015.^19^ After developing a mouse model with PROS/CLOVES and demonstrating the prevention and improvement of overgrowth with the use of BYL719, Venot et al. treated 19 patients (four adults and 15 children) with PROS under a compassionate care protocol. All patients had symptom improvement and measurable radiologic and clinical tumor decrease. The authors reported no significant adverse effects, but some patients developed hyperglycemia that was controlled with diet. BYL719 appears to act by decreasing AKT phosphorylation and decreasing mTORC1 activation. Venot and colleagues suggested that the beneficial effect of BYL719 in this patient population was driven by more complete blockage of AKT in comparison to sirolimus. PIK3CA inhibition is a promising treatment for patients with PROS who overall have high morbidity and mortality, particularly those who have suboptimal or no response to sirolimus. A retrospective chart review study of patients with PROS who have received alpelisib through the Novartis Managed Access Program is in process. A double-blinded, placebo-controlled clinical trial assessing the efficacy, safety, and pharmacokinetics of alpelisib in pediatric and adult patients is underway (NCT04589650).

### 4.4 | MEK inhibitors

MEK inhibitors are drugs that inhibit either MEK1 or MEK1 and 2, the mitogen-activated protein kinase kinase (MAPK) enzymes. Several MEK inhibitors are FDA-approved for treatment of melanoma. The MAPK pathway consists of Ras, Raf, MEK, and ERK signaling proteins, and mutations in genes encoding this pathway have been implicated in AVMs, vascular tumors, complex VAs, and CCLA.^20–25^

Preclinical data support the use of MEK inhibitors in a zebrafish model and mouse model of AVMs and zebrafish model of lymphatic anomalies.^23,25,26^ Several groups have also reported successful use of MEK inhibitors in patients with either AVMs or lymphatic anomalies with documented somatic mutations in the MAPK pathway. A patient with a large AVM of her trunk found to have a somatic MAP2K1 mutation responded to treatment with trametinib with decrease in size, color, and warmth of the lesion.^27^ A patient with spinal arteriovenous metameric syndrome with a somatic KRAS mutation was treated with trametinib with resultant decreased arterial inflow to the malformation.^28^ Additionally, a patient with capillary malformation-AVM syndrome with high-output cardiac state and a EPHB4 mutation was successfully managed with trametinib.^29^ Three patients with severe lymphatic disorders, a male with CCLA due to ARAF mutation, a female with Noonan syndrome and SOS1 mutation with protein-losing enteropathy and GI bleeding, and a young woman with KLA and a CBL mutation, were treated with trametinib with marked results. These patients had significant symptomatic improvement as well as radiographic response with restructuring of the lymphatic system.^30^

Trametinib doses in these cases range from 0.5 to 1 mg per day. Dosing for trametinib in VAs has been based on phase I trials for pediatric solid tumors, plexiform neurofibromas, and JMML, using 0.025 mg/kg/day. Dosing in adults varies from 0.5 to 2 mg daily. Patients with VAs may need lower doses, but dosing for MEK inhibitors and VAs needs to be investigated further. Monitoring during therapy includes laboratory evaluation with liver function tests, echocardiography, and ophthalmologic exams, as well as clinical monitoring for pulmonary dysfunction and dermatologic effects. Common side effects include acneiform rash, palmar-plantar erythrodysethesia, paronychia, and other cutaneous infections. Supportive care requires intermittent treatment of the dermatologic side effects. Two phase II trials, NCT04258046 in the United States and EudraCT 2019-003573-26 in Belgium, will prospectively evaluate the use of trametinib in AVMs. The most common MEK inhibitor described thus far has been trametinib, but other MEK inhibitors may be used in future patients and trials.

## 5 | OTHER MEDICAL THERAPIES

### 5.1 | Antiangiogenic medications

Thalidomide has antiangiogenic properties mediated by downregulation of VEGF expression. It is used as a therapy for hereditary hemorrhagic telangiectasias (HHT), and recently has been studied in a trial of patients with life-threatening stage III or IV facial AVMs, with improvement in bleeding, ulceration, pain, and deformity.^31^ The dose in the prospective trial for AVMs ranged from 50 to 200 mg daily.

Thalidomide carries a black box warning for pregnancy and thromboembolic events, and requires prescription through a restricted distribution Risk Evaluation and Mitigation Strategies (REMS) program. Monitoring during therapy includes laboratory evaluation with complete blood count, thyroid function tests, and pregnancy test, and clinical monitoring for signs of neuropathy. Peripheral neuropathy occurs in over 10% of patients and may be irreversible. Other antiangiogenic medications such as bevacizumab have been used in patients with HHT and hepatic AVMs and patients with brain AVMs.^32,33^

### 5.2 | Combination therapies

Some patients with severe manifestations of disease or those not responsive to monotherapy may benefit from combination therapies. In vitro studies in osteosarcoma models demonstrate a synergistic effect between zoledronic acid and everolimus in decreasing PI3K/mTOR signaling, and a patient with GSD who was refractory to zoledronic acid and had only modest response to sirolimus monotherapy showed significant improvement to treatment with combination zoledronic acid and sirolimus.^34,35^ Subsequent multicenter retrospective review of 25 patients with complex lymphatic anomalies, including GSD, GLA, and CCLA, found the combination of sirolimus and bisphosphonates to be an effective therapy.^36^

Hematologic derangements such as KMP or consumptive coagulopathy complicate some vascular tumors and malformations and may prove life-threatening, warranting combination therapy. An infant with KMP in the setting of KHE had rapid improvement in his tumor size and transfusion dependence after the addition of sirolimus to his regimen of vincristine and prednisolone.^37^ Additionally, a patient with KLA and coagulopathy treated with prednisone, sirolimus, vincristine, and zoledronic acid experienced clinical improvement, normalization of hematologic parameters, as well as normalization of the plasma biomarker angiopoietin 2.^38^

Combination therapies for patients who are refractory to monotherapy or require multimodal therapy for severe complications may provide synergistic effects. A mouse model of venous malformation showed regression of disease in response to therapy with an ABL kinase inhibitor in combination with sirolimus. This work may portend success for future clinical application of combination therapy for these diseases.^39^ A caveat is that combination therapy will likely cause more side effects, and a more robust understanding of the mechanism of action of these agents is essential. Use of combination therapies should be in the setting of severe or refractory disease and should be directed by a VA specialist.

## 6 | DISCUSSION

The successful treatment of complex VAs with sirolimus starting in 2010 catapulted the field forward and left us poised to approach a wide range of vascular malformations and tumors with targeted medical therapies. Experience with targeted therapies in solid tumors and leukemia and training in monitoring adverse events and the use of off-label therapies or drugs in early phases of trials uniquely equips pediatric hematologists/oncologists to treat patients with VAs. Recent advances in the understanding of the genetic underpinnings of VAs have broadened the potential therapies for these patients, but many implementation issues remain.

Optimal response criteria in this population remain a critical area for future study. Oncologic response criteria are not broadly applicable in patients with VAs, as the goal of treatment of VAs is not complete disease eradication. Treatment goals for patients with VAs may include reduced malformation size, stabilization of growth, normalization of coagulation parameters, decrease in lymphatic leakage, reduced rate of infections, improved mobility, or overall improvement in health-related quality of life. Quantifying response, either with volumetric imaging, change in laboratory parameters, anthropometric measurements, functional measures, or documentation of frequency of complications such as bleeding or infection, provides physicians and patients important assessments of the efficacy of therapy. Use of biomarkers may be an important adjunct in ascertaining response to targeted therapy.

Future drug discovery will be important as we consider the diverse array of genotypes in patients with VAs. Potential emerging therapies in this arena include TIE2 inhibitors for venous malformations, many of which are driven by mutations in TEK, as well as RAS inhibitors in the growing number of lymphatic disorders found to be driven by RAS mutations. These agents are already in development for patients with cancer.

As the possibilities for medical treatment of VAs expand, many questions remain regarding optimal duration of therapy, strategies for discontinuation, long-term use of these medications, and potential late effects, all of which are vital for future study in order to optimize care of these patients. Remembering a history in which patients with VAs had few therapeutic options, we look forward to ongoing multidisciplinary collaboration to overcome the often severe complications and negative effects on quality of life by maximizing medical therapies for patients with VAs.

## Figures and Tables

**TABLE 1:** Pathway alterations underlying vascular anomalies and potential targeted therapies

Pathway	Representative diagnoses	Genetic mutations	Targeted therapies	Level of evidence
PI3K/AKT/mTOR pathway	Blue rubber bleb nevus syndrome	*TEK*	PIK3CA inhibitors (alpelisib)	Preclinical data^19^ Case series^19^
	CLOVES syndrome	*PIK3CA*		Clinical trials ongoing
	Fibro adipose vascular anomaly	*PIK3CA*		
	Generalized lymphatic anomaly	*PIK3CA*	AKT inhibitors	Preclinical data^16^
	Klippel–Trenaunay syndrome	*PIK3CA*	(miransertib)	Case reports^18^
	Lymphatic malformations	*PIK3CA*		Clinical trials ongoing
	PTEN hamartoma syndrome	*PTEN*		
	PIK3CA-related overgrowth syndrome	*PIK3CA*	mTOR inhibitors	Phase II clinical trial^3^
	Proteus syndrome	*AKT1*	(sirolimus)	Phase III trial ongoing
	Venous malformations	*TEK, PIK3CA*	(everolimus)	Case reports^40^

RAS/MAPK/ERK pathway	Arteriovenous malformations	*KRAS, MAP2K1, BRAF*		
	Capillary malformation-arteriovenous malformation	*RASA1, EPHB4*		
	Central conducting lymphatic anomaly	*ARAF, SOS1, PTPN11*	MEK inhibitors (trametinib)	Preclinical data^23,25,26^ Case reports^27–30^ Clinical trials ongoing
	Hereditary hemorrhagic telangiectasia	*RASA1*		
	Kaposiform hemangioendothelioma	*GNA14*		
	Kaposiform lymphangiomatosis	*NRAS*		
	Tufted angioma	*GNA14*		

VEGF pathway	Hereditary hemorrhagic telangiectasia	*ENG*	Thalidomide Lenalidomide	Phase II clinical trials^41^
		*ACVRL1*	VEGF inhibitors (bevacizumab)	Phase II clinical trial^32^ Phase III trial ongoing
		*SMAD4*	VEGFR inhibitors (pazopanib)	Case series^42^

Abbreviation: CLOVES- congenital lipomatosis, overgrowth, vascular malformations, epidermal nevi, spinal/skeletal anomalies, scoliosis.

## Abbreviations:

AVM: arteriovenous malformation
CLVM: capillary lymphatic venous malformations
CCLA: central conducting lymphatic anomalies
CLOVES: congenital lipomatosis, overgrowth, vascular malformations, epidermal nevi, spinal/skeletal anomalies, scoliosis
GLA: generalized lymphatic anomaly
GSD: Gorham–Stout disease
HHT: hereditary hemorrhagic telangiectasias
KHE: kaposiform hemangioendothelioma
KLA: kaposiform lymphangiomatosis
KMP: Kasabach–Merritt phenomenon
mTOR: mammalian target of rapamycin
MAPK: mitogen-activated protein kinase
PI3K: phosphoinositide-3-kinase
PROS: PIK3CA-related overgrowth syndromes
PJP: *Pneumocystis jirovecii* pneumonia
VA: vascular anomaly
